# Empirical Study of User Preferences Based on Rating Data of Movies

**DOI:** 10.1371/journal.pone.0146541

**Published:** 2016-01-06

**Authors:** YingSi Zhao, Bo Shen

**Affiliations:** 1 School of Economics and Management, Beijing Jiaotong University, Beijing, 100044, China; 2 School of Electronic and Information Engineering, Key Laboratory of Communication and Information Systems, Beijing Municipal Commission of Education, Beijing Jiaotong University, Beijing, 100044, China; IFIMAR, UNMdP-CONICET, ARGENTINA

## Abstract

User preference plays a prominent role in many fields, including electronic commerce, social opinion, and Internet search engines. Particularly in recommender systems, it directly influences the accuracy of the recommendation. Though many methods have been presented, most of these have only focused on how to improve the recommendation results. In this paper, we introduce an empirical study of user preferences based on a set of rating data about movies. We develop a simple statistical method to investigate the characteristics of user preferences. We find that the movies have potential characteristics of closure, which results in the formation of numerous cliques with a power-law size distribution. We also find that a user related to a small clique always has similar opinions on the movies in this clique. Then, we suggest a user preference model, which can eliminate the predictions that are considered to be impracticable. Numerical results show that the model can reflect user preference with remarkable accuracy when data elimination is allowed, and random factors in the rating data make prediction error inevitable. In further research, we will investigate many other rating data sets to examine the universality of our findings.

## Introduction

User preferences are considered to be the user’s opinions on social topics, goods, services, friends, works, ads, the search results of search engines, and more. Ordinarily, user preferences are closely related to recommender systems, because the task of a recommender system is to convert data on users and their preferences into predictions of their possible interests [1, 2]. Although recommender systems are not the only places to display the prowess of user preferences, they generate a heavy demand for user preferences, and create huge amounts of data, which provides the opportunity to mine and learn more characteristics of the user preferences.

In the recommender system field, researchers mainly focus on how to improve the accuracy of recommendations [3–8], which implicitly involves how to obtain user preferences. One important method is collaborative filtering (CF) [9]. CF is based on the fact that people make their decisions about new things based on their own knowledge history, as well as the experiences of other related people [2], e.g., as expressed on Amazon’s website: “Customers Who Bought This Item Also Bought.” Of course, in a recommender system, CF is considered to be a kind of data filtering algorithm. In CF models, the key issue is how to measure the similarity between users [10–12] or between items [3, 12, 13], which directly concerns the degree of correlation between the analyzed target and other reference objects. The common methods of similarity measurement include overlap [14], Euclidean distance [15], Hamming distance [16], Pearson correlation [17], and the cosine of the angle between vectors [18]. There are also many improved and adjusted methods [1, 2, 18] based on traditional metrics in the literature. Each of these methods has its own advantage, and no method wins out over all others. However, it is commonly recognized that the similarity between items tends to be more static than the similarity between users [1]. Model-based methods are also available, which include SVD [19], LSA [20], Bayesian [21], fuzzy [22], and neural networks [23]. These methods aim to directly calculate recommendations through pre-created models rather than by obtaining the relation between users or items. Thus, user preferences are hidden in models. Some of these models are also used to reduce the dimensionality of the data, such as SVD and LSA. In addition, they usually have higher commendation accuracy. Because of the absence of explicit physical meanings about user preferences, it is usually difficult to improve these methods and to understand how users make decisions by them.

Whether CF or model-based methods are used, history data about users and items are the basis. There are two kinds of data: two-valued data and multiple-valued data. Two-valued data only convey “like” and “dislike” opinions from users about an item. Multiple-valued data contain the ratings of users for items, which in general are integers with a range of 1–5. Ratings can be regarded as a kind of reflection of user preferences on the dimension of a certain object. For simplicity, some researchers map multiple-valued data to two-valued data, especially when the purpose of the study is to find general rules about user preferences [14, 24].

Although more and more factors are being included in recommender systems, and new algorithms are continually presented, what affects a user’s decisions and whether it can be predicted accurately are still open issues. In particular scenarios, other researchers attempt to determine the key factors that affect user preferences. Ref [24] presented a weighting method to extracting the hidden information of networks formed by users and items. By assigning a heterogeneous distribution of initial resources [16] and removing the redundant correlations [25], the original method and its improved methods find several factors related to user preferences. In Ref [14], statistical methods were used to explore affinity relations. The authors found that there was an intrinsic limit, which would prevent the achievement of perfect prediction by statistical means, even if more data were obtained.

From another viewpoint, the development of computational social science makes it possible to study human behavior using online data [26]. The authors of Ref. [27] found that, through analyzing massive data, a better understanding of collective human behavior could be achieved, and more evidences were presented in [28]. Further, the research of [29] indicated that users’ collective future behavior can be predicted by what they searched for online. The research on social opinion has some goals in common with recommender systems. For example, researchers want to know how people format or change their opinion about a given topic [30]. Many theories have been developed, such as the majority rule model [31], social impact theory [32], and bounded confidence model [33], which could also be considered to be methods for understanding user preferences. However, unlike the study of social opinion, recommender systems do not consider the macroscopic state and the evolution process of user preferences.

In this paper, we introduce the results of an empirical study of user preferences based on rating data. We first analyze the relationships between users and items, and then map them into a hyper-network. We present a kind of distance measure method, and find some interesting characteristics about user preferences. Based on our findings, we propose a user preference model, which employs the relations between items and a user’s history ratings to evaluate their preference for new items. We also discuss the results of the proposed model. It should be noted that in this paper, we only take into account the empirical study of a user’s preference using a special data set, instead of building a recommendation algorithm or a recommender system.

## Empirical Analysis

In this paper, we use one of the standard benchmark data sets, namely *MovieLens* [34], to carry out our analysis. The data set we used contains 100,000 ratings by 943 users on 1,682 movies. Each rating item is an integer in the range of 1–5. In one example, listed in Table 1, users u_1_, u_2_, u_3_, and u_4_ provide ratings for movies a, b and c. These ratings can be regarded as a kind of relationship between the users and movies.

**Table 1:** Relationship between users and movies based on ratings.

	a	b	c
u_1_	r_1a_	⌀	⌀
u_2_	r_2a_	r_2b_	⌀
u_3_	⌀	r_3b_	r_3c_
u_4_	r_4a_	⌀	r_4c_

**Fig 1:**
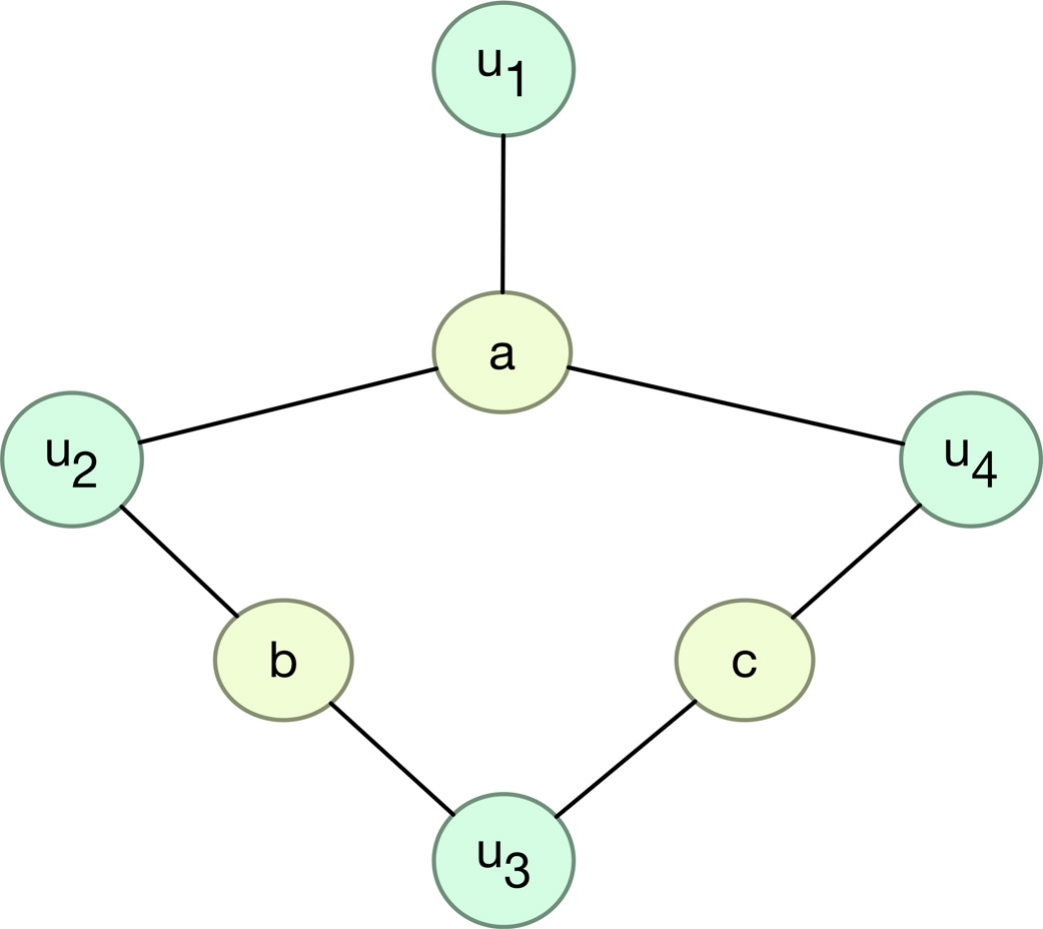
Network that is composed of user nodes and movie nodes. Movie ratings from users are the connections between users and movies.

**Fig 2:**
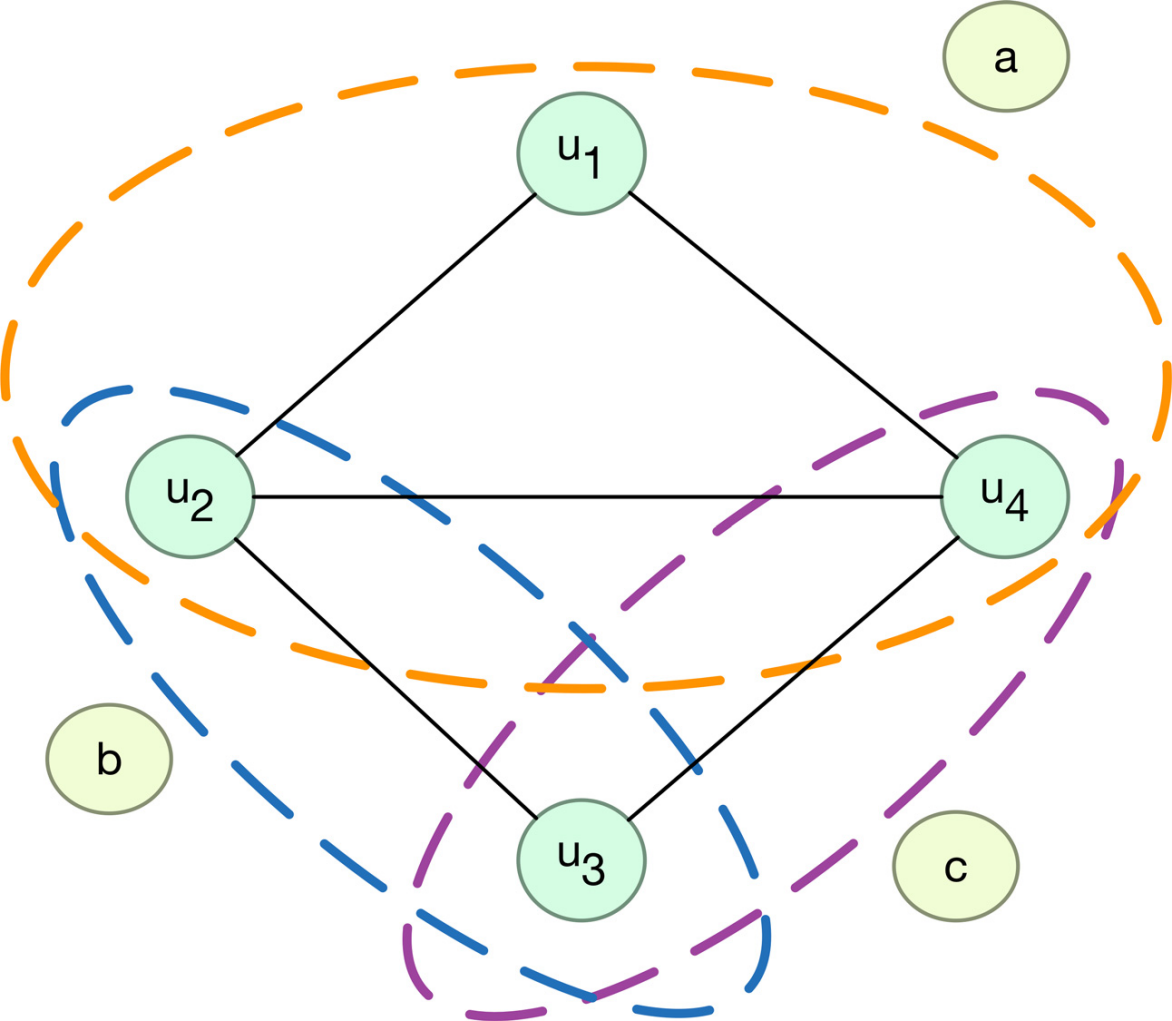
Hyper-network in which user is treated as node and movie as hyper-edge.

The relationship can also be presented in the form of a network, as shown in Fig 1. From the viewpoint of the network, the nodes of movies connect users together, and the nodes of users connect movies together. Obviously, there are two kinds of different nodes in these networks, user nodes and movie nodes. If the movie nodes are extracted, the network will have the structure shown in Fig 2, which is a so-called hyper-network [35–37].

A hyper-network consists of pairs *H* = (*V*, *E*), where *V* = {*v*_1_, *v*_2_,⋯,*v*_*n*_} is the set of nodes, and *E* = {*e*_1_, *e*_2_,⋯,*e*_*m*_} is the set of hyper-edges, with *e*_*i*_ ∈ *V* for *i* = 1,2,⋯,*m* [37]. Clearly, in a hyper-network, each hyper-edge is a subset of the set of nodes and contains at least two nodes, as illustrated in Fig 2. Here, each movie is a hyper-edge, e.g., movie a is associated with users {u_1_, u_2_, u_4_}, b with {u_2_, u_3_}, and c with {u_3_, u_4_}. In addition, we can deem that the nodes belonging to a hyper-edge fully connect to each other.

From the perspective of the hyper-edge, a hyper-network can be defined as a set of *R*, which is the relation between two sets *A* and *B* [35]:

R(a)={b|b∈B,a→b} (1)

$$H_{A}(R,B)=\{R(a)|a\in A\}$$ (2)

**Fig 3:**
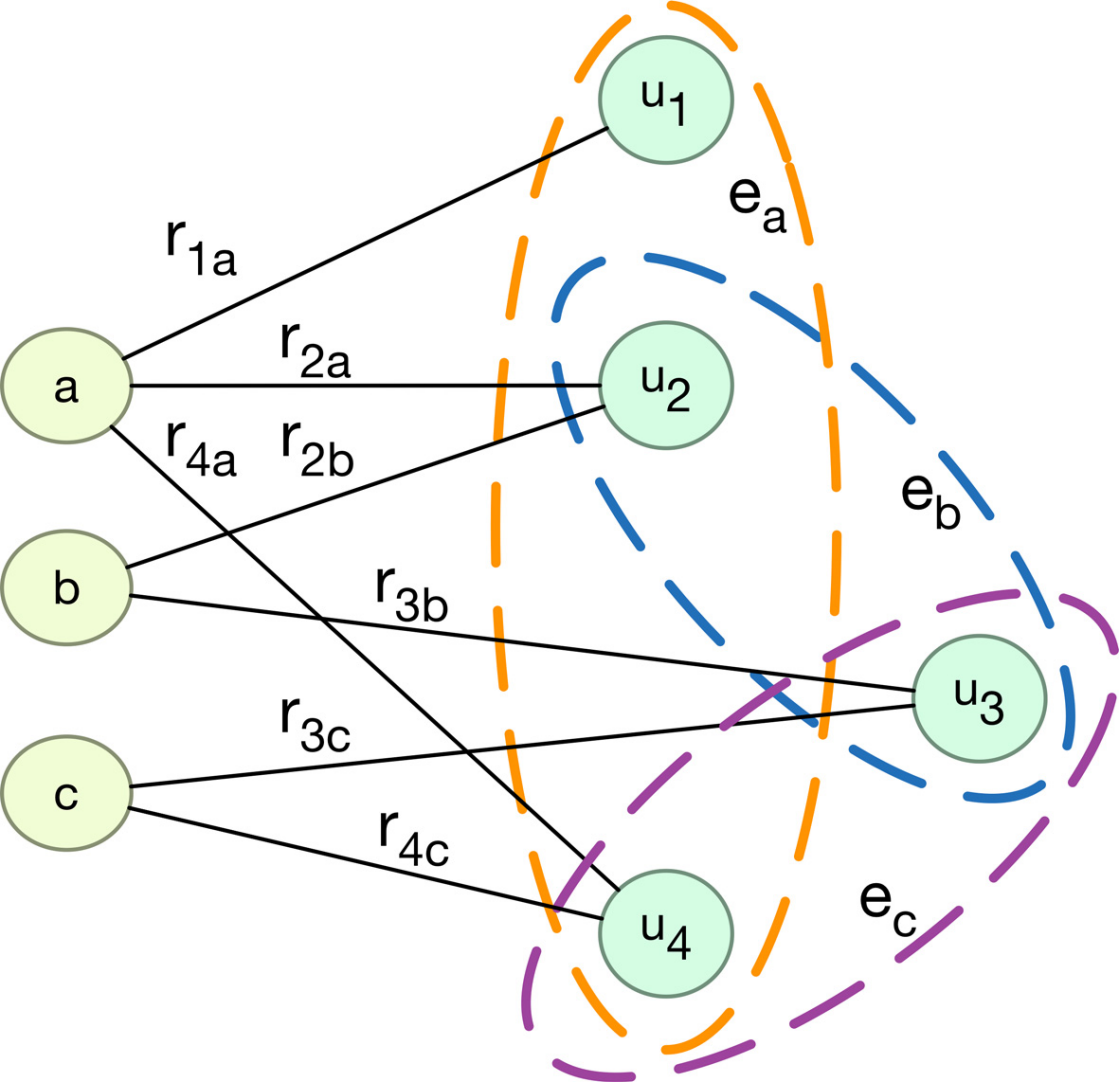
Bipartite hyper-network that is constructed by movie set, user set and the relation between users and movies. r_ix_ is the rating that user *i* votes for movie *x*.

Here, *a* → *b* means distinctly that *a* relates to *b*. Let all movies be set *A*. Let all users be set *B*, and let all ratings be the relation between *A* and *B*. Then, the network in Fig 2 can just be mapped into a bipartite hyper-network, as shown in Fig 3. In this bipartite hyper-network, movies correspond to hyper-edges, e.g., movie *a* corresponds to hyper-edge *e*_*a*_, i.e., *R*(*a*), which is a subset of user set *B*. A bipartite network can be used to describe many-to-many relations with two object sets in the real world, such as a flavor network [38], scientific collaboration network [39], users and products network [24] and so on. Many researchers employ bipartite networks as a tool to study relations [40].

From the hyper-edge viewpoint, the estimation of how a user will like a movie can be converted, to some extent, into finding what correlation exists between the hyper-edges that the user belongs to and the hyper-edge that the user will belong to. For example, if we need to predict the opinion of user u_3_ in Fig 3 about movie a, the correlations between hyper-edge *e*_*b*_ and *e*_*a*_, and between *e*_*c*_ and *e*_*a*_, may provide useful information.

The hyper-edge characteristics can usually be modeled by employing the concept of the simplex volume because a hyper-edge is regarded as a simplex [41]. However, simplex volumes degenerate when the degree of the hyper-edge is larger than the dimensions of the feature [42, 43], which is exactly the case when treating movies as hyper-edges.

We define the distance between two hyper-edges *e*_*i*_ and *e*_*j*_ as follows.

$$d_{ij}=\alpha_{ij}\sqrt{\varphi_{ij}\sum_{k\in R(i)\cap R(j)}{\left(\frac{r_{ik}-r_{jk}}{s_{k}}\right)}^{2}}$$ (3)

$$\alpha_{ij}={\left(\left|R(i)\cup R(j)\right|/\left|R(i)\cap R(j)\right|\right)}^{\frac{1}{2}}$$ (4)

$$\varphi_{ij}={\left(\left|R(i)\cap R(j)\right|\right)}^{-1}$$ (5)

where *r*_*xy*_ is the rating that user *x* gave to movie *y*, *s*_*k*_ is the standard deviation between *r*_*ik*_ and *r*_*jk*_, and |*X*| denotes the number of elements in hyper-edge *X*.

*φ*_*ij*_ is called the shrinking factor and is used to eliminate the cumulative effect of the standardized difference between two ratings. *a*_*ij*_, called the stretching factor, is designed to reflect the extent that the two hyper-edges overlap their union. Obviously, when *R*(*i*) = *R*(*j*), we have *a*_*ij*_ = 1, and if *R*(*i*) ∩ *R*(*j*) = ∅, then *a*_*ij*_ = +∞. This seems a reasonable measurement of the correlation between movies represented by hyper-edges. When more users gave them the same ratings, the more common characteristics they could have.

**Fig 4:**
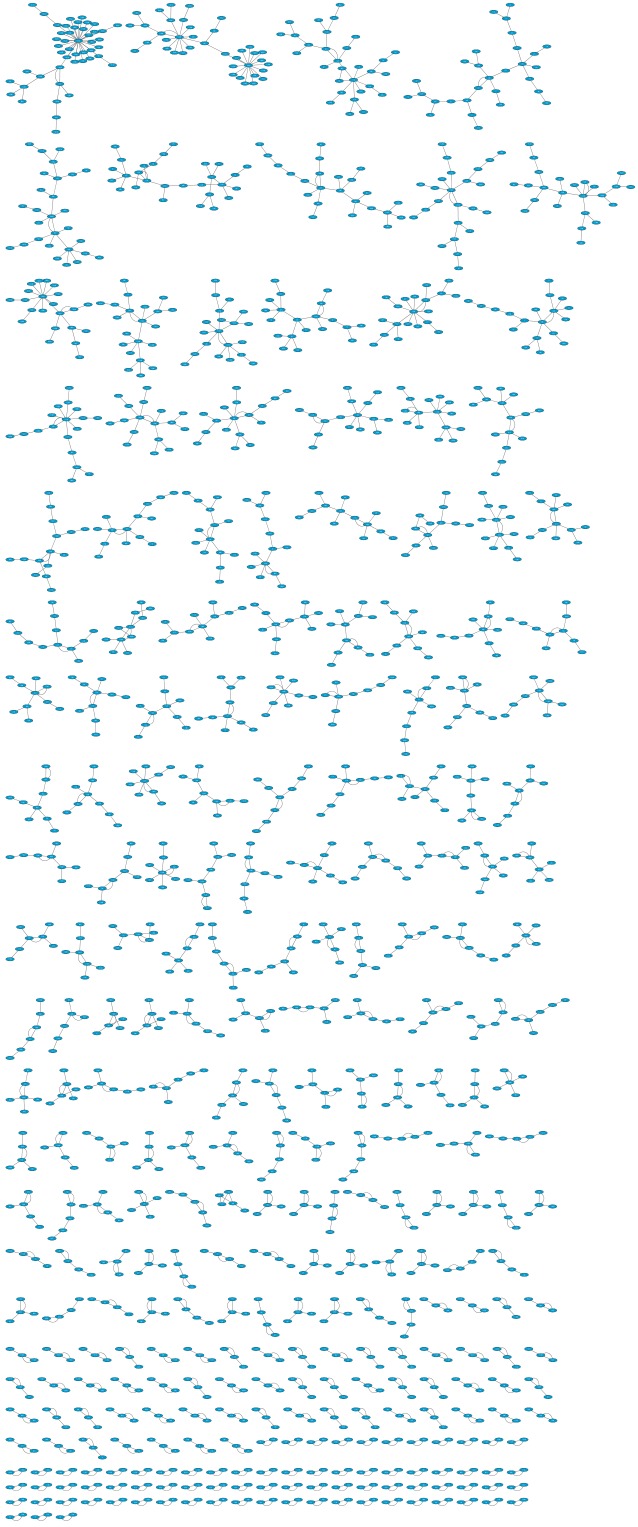
Cliques in which each node only connects to its first-order h-neighbors, plotted by Crytoscape [44].

We calculated the distance between any two movies in the *MovieLens* data set using Eq 3. The distance data were stored in S1 File. Fig 4 plots the network of data set u.data by Crytoscape [44]. In the plot, each node represents one movie, and a movie only has one connection to its first-order nearest neighbor (S2 File) in the sense of distance defined by Eq 3. For simplicity, we call the nearest neighbor the h-neighbor and the connecting relation h-connected.

The results show that these movies form many sub-networks (named cliques here) with different sizes, and there is no connection between these cliques (286 cliques for data set u.data). This implies that the movies in the dataset have the potential characteristic of closure, which could be the result of users’ selections with explicit preferences. Then, the closure feature of the cliques could be used to evaluate the preferences of users who have voted for some of the movies in a clique. Similar clique structure also appears on other networks, such as Flickr and CiteUlike [45].

**Fig 5:**
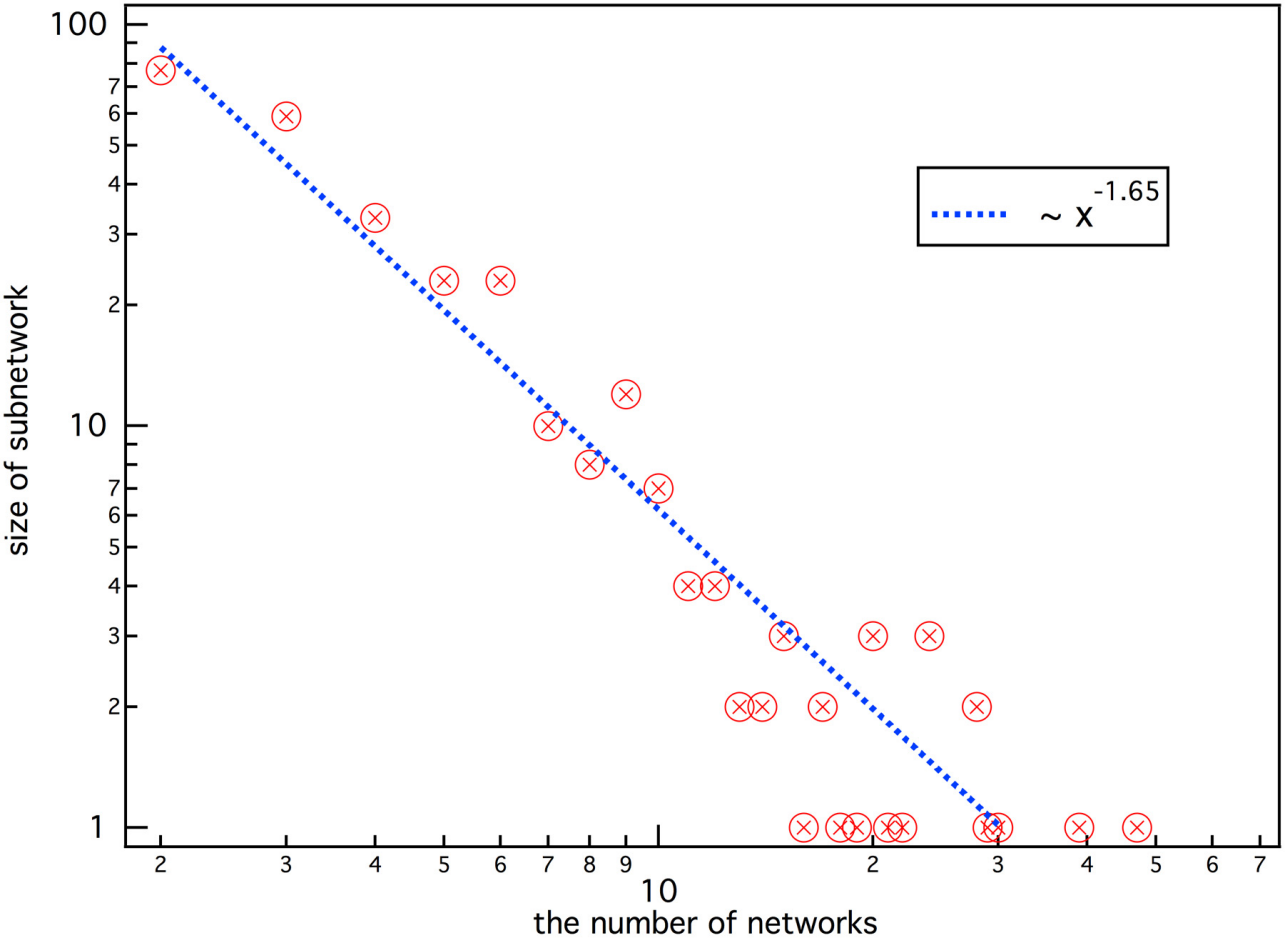
The size of cliques vs. the number of corresponding networks.

Furthermore, we also notice that only a small number of cliques contain a large number of nodes, while most have only a few nodes under the condition of first-order h-neighbors. We plot the statistical results in Fig 5, which shows that the distribution of the clique sizes closely follows a power law: *S*(*x*) ∼ *x*^−*τ*^, whereτ is a constant exponent with a value of about 1.65. Similar phenomenon was also observed in many other real systems which can be modeled as bipartite networks [1]. For example, the item-degree distributions of the e-commerce data in amazon.com [46], the music sharing data in audioscrobbler.com [47] and the movie data in the Internet Movie Database [48] all obey power-law-like form with different exponent value.

One possible explanation for this power law is that the numbers of ratings received by the movies are inhomogeneous. Because the data in the movie data set was collected during a short period of seven months, new movies at that time evidently received more ratings, while old movies got less attention. Although every user gave at least 20 ratings, about 44.8% of the movies had less than 20 rating, and about 79% of the movies had less than 94 ratings, which is 1/10 of the number of users.

When connecting movies by first-order h-neighbor, those movies that had fewer ratings would choose the h-neighbor in a greater range. Thus, more nodes connected together, and few large networks formed. We would expect the size of the cliques to become more homogeneous when data could be retrieved over a long time range. However, even in the data for a prolonged time period, the differences between users and the differences between movies will still lead to various cliques.

We also notice that there are many pairs of nodes, which are the first-order h-neighbors of each other, and each clique has one pair of such nodes, expressed as Λ(1) = {(*α*, *β*)|*α* ∼ *β*, *α*, *β* ∈ *H*_*A*_}. If a clique has only two nodes and they are Λ(1), we call it the first-order h-neighbor clique. This indicates that some common characteristics bring them together with a stronger connection, which may cut off their relations to other nodes when the first-order h-neighbor rule is applied. For example, the nodes representing the movies *Batman Forever* (1995) and *Batman Returns* (1992) connect together to form a clique with two nodes. They are the h-neighbors of each other. Under the rule of the first-order h-neighbor, the existence of these nodes is the reason for the closure of the clique.

**Fig 6:**
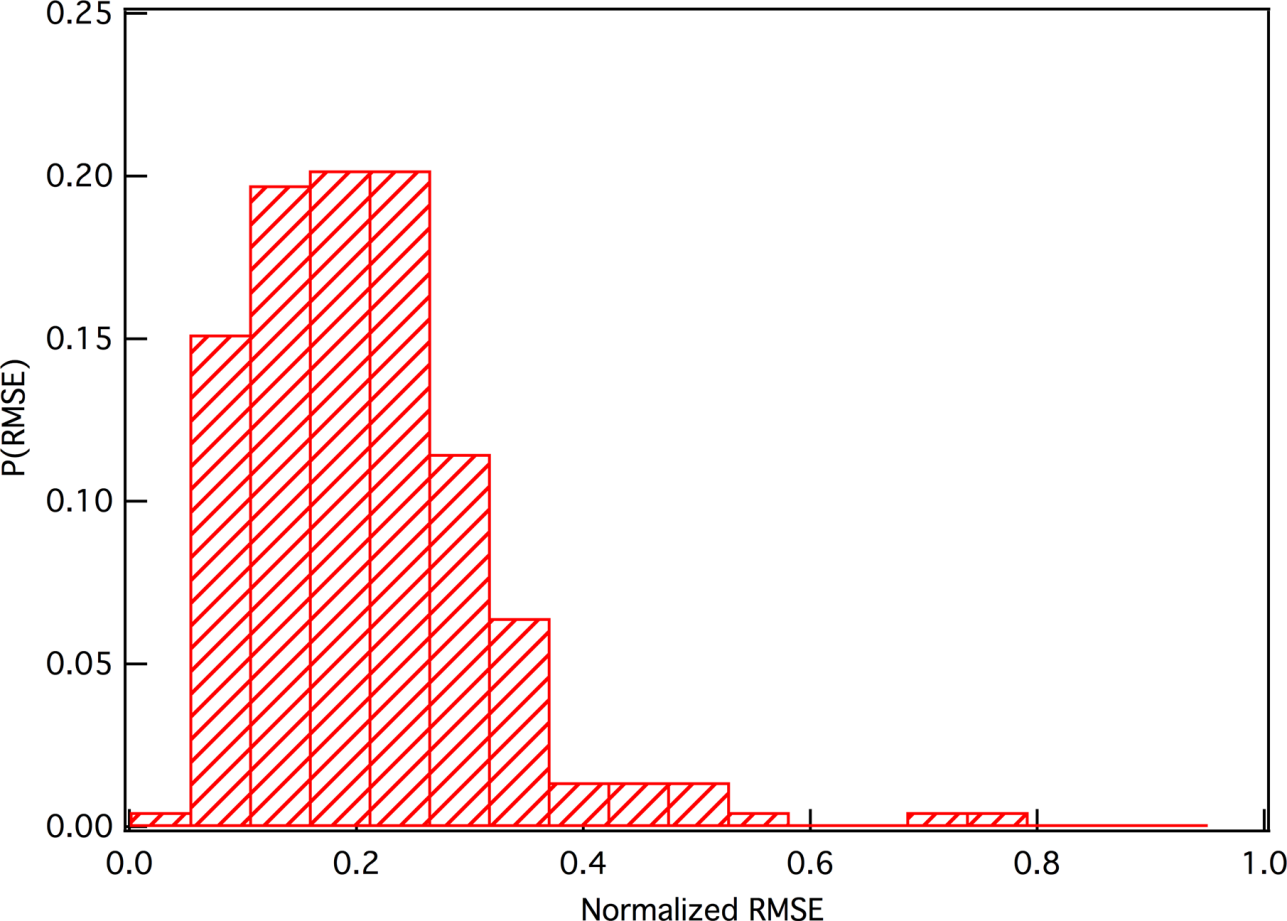
The distribution of RMSE of ratings that users voted for the first-order h-neighbor clique.

We found the statistics for all the ratings of the movies that belong to Λ(1) using the following method:

$$P(\varepsilon )=\sum_{\kappa }\delta (\varepsilon_{\kappa },\varepsilon )/\;N,\;\kappa \in \Lambda (1)$$ (6)

where *ε*_*k*_ is the normalized RMSE of the ratings users gave to a pair of nearest nodes, *N* is the number of the pair, and *P*(*ε*) is the distribution of the normalized RMSE. *δ* is the Kronecker symbol. The results are plotted in Fig 6. Clearly, 75% of the normalized RMSE values lie in the range of 0.05–0.35. The maximal RMSE is 4.0. Thus, the ratings that users gave to the first-order h-neighbor nodes have RMSE values of 0.2~1.4, and most are less than 1.0. This means that most users have similar opinions about Λ(1). That is to say, from the viewpoint of the users, these two movies show a strong similarity. It should be further emphasized that here the h-neighbor nodes forming a clique are movies. Although the first-order h-neighbor clique implies these movies have similarity and a user related to these movies has similar opinions on them, it does not mean that these users related to a clique have the same preference on different types of movies. It should also be noted that here the RMSE value is not comparable with that used in predicting precision, because it is calculated for two different movies.

**Fig 7:**
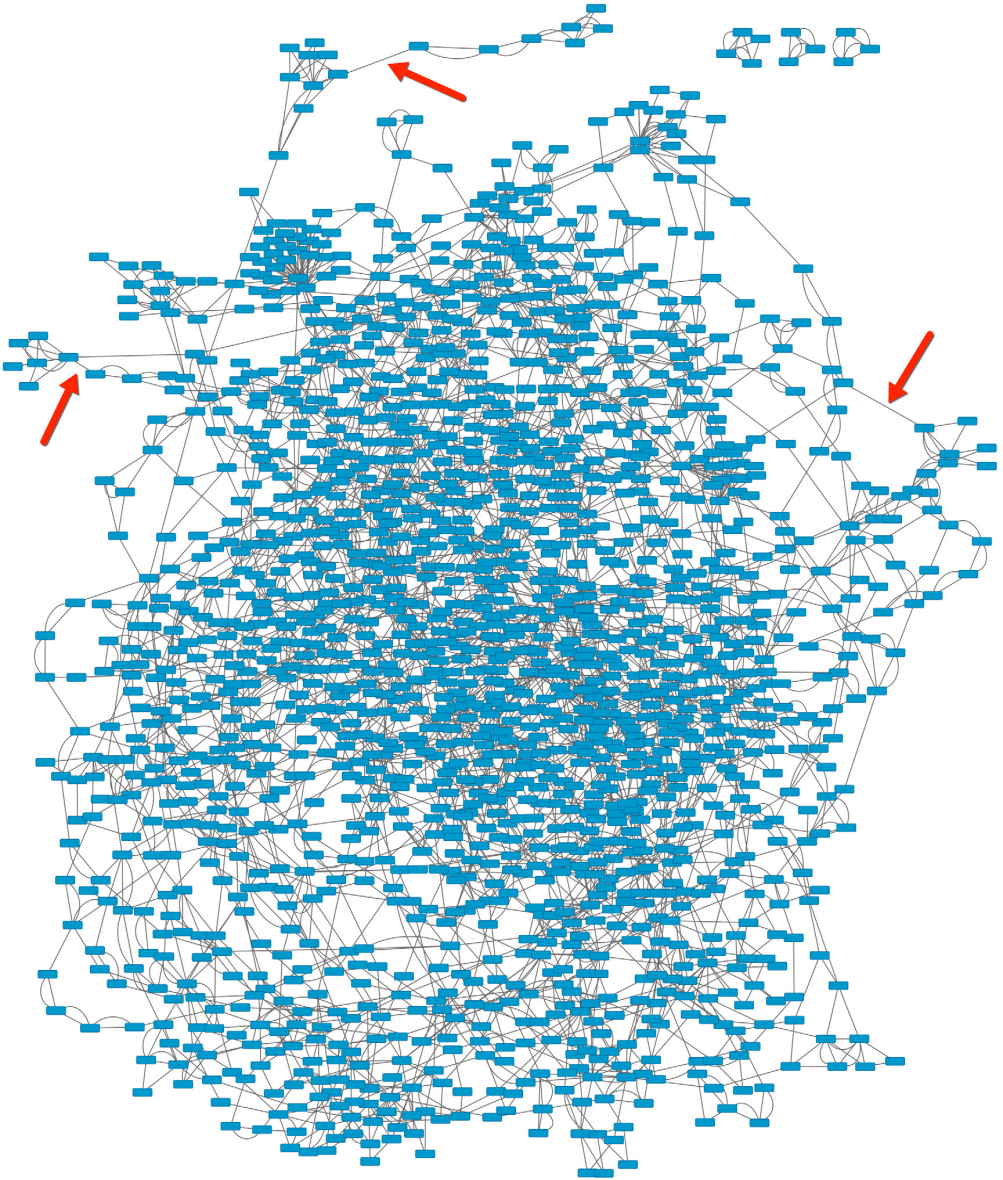
Cliques in which each node only connects to its second-order h-neighbors, plotted by Crytoscape [44].

We further measure the network constructed by the second-order h-neighbor rule (S3 File), in which the first-order h-neighbors are included. In this case, the closure characteristic almost disappears, as shown in Fig 7. The detailed data indicate that the diversity of the distance increases under the condition of the second-order h-neighbor rule, which causes more nodes to connect together. In other words, the second-order h-neighbors make connections between the nodes that are discrete under the first-order neighbor rule, as shown by the links indicated by the red arrows in Fig 7.

Obviously, the second-order h-neighbor rule blurs the edge of the cliques formed by the first-order h-neighbor rule. Although more nodes are connected into cliques, the similarity between the nodes in a clique is reduced. Thus, using a distance threshold may be a better idea to keep the closure and avoid the diverse distance effect. We will study this in more depth in the future.

**Fig 8:**
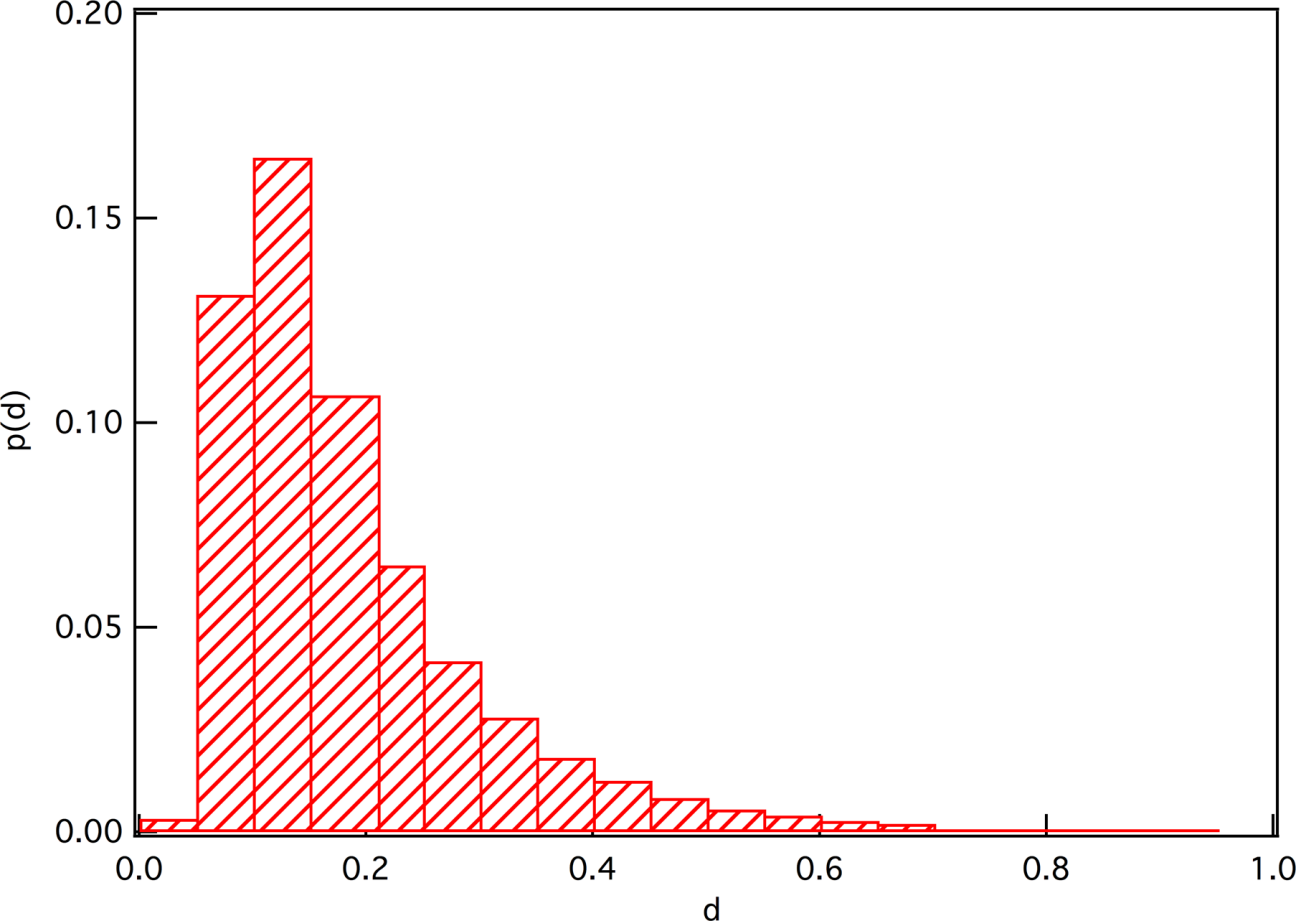
The distribution of distance between movies.

We also investigate the distribution of the distance between movies using the method reported in ref. [14] with Eq 7. The distribution plot is shown in Fig 8.

$$P(d)=\sum_{i}\sum_{j\neq i}\delta (d_{ij},d)/\;N(N-1)$$ (7)

where *δ* is the Kronecker symbol, *N* is the number of movies, and *d*_*ij*_ is the distance between movies *i* and *j* by Eq 3.

Clearly, the distance is a rather homogenous distribution as a result of *P*(*td*) = *t*^−1^ *p*(*d*). This is essentially in agreement with the result of ref. [14] which was achieved on the *EachMovie* data set, except that the distribution of the distances between movies does not appear to be polarized. Moreover, its peak is less than 0.2 and around *d* ∼ 0.17, which means, according to the result of ref. [14], that we would be able to use the information contained in the relations between movies to describe user preferences and predict their ratings. It also means that we do not need the information about the similarity between users.

## User Preference Model and Results

In the above empirical analysis of the *MovieLens* data, we found that the hyper-network of movies shows the characteristic of closure under the condition of considering only the first-order *h-connected*, and the sizes of these closure cliques demonstrate a power law distribution, which reflects the existence of some interdependency between some movies, and users’ opinions about movies have potential tendencies. The distribution of the distance between any two movies gives further evidence that the relations between movies can be used to describe the preferences of users.

The basic idea is to use the information about the relations between movies to estimate users’ opinions: if we want to know the opinion of user *i* about movie *a*, we could use the opinion of user *i* about movie *b* that is a first-order *h-neighbor* of movie *a* for the estimate.

However, there are still two obvious issues to be considered:

1. Many first-order *h-connected* cliques are too small.
2. Lots of movies have fewer ratings.

For a clique with a small size, if user *i* rated movie *a*, then predicting the rating that user *i* will give to movie *b* is reasonable when *a* and *b* are first-order *h-neighbors*. In contrast, if user *i* did not rate any movie in a clique, the prediction for the movies in this clique will become unreasonable.

In consideration of the above empirical analysis results and to overcome these issues, we present a user preference model, as follows:

$${ϒ}_{i}(\beta )=\frac{\sum_{x}r_{ix}d_{x\beta }^{-1}}{\sum_{x}d_{x\beta }^{-1}},\;x\;\tilde{\in }\;M=\left\{m|\underset{m,\;k}{\text{arg max}}(d_{m\beta })\right\}$$ (8)

where ϒ_*i*_(*β*) denotes the estimation of the opinion of user *i* about movie *β*, *r*_*ix*_ is the rating that user *i* gave movie *x*, and *d*_*xβ*_ is the distance between movies *x* and *β*. $\left\{m|\underset{m,\;k}{\text{arg max}}(d_{m\beta })\right\}$ is the set that contains the nearest *k* movies to movie *β*, where *k* is a tunable parameter. $\tilde{\in }$ means taking members contiguously.

Obviously, the user preference model employs more than one movie and their ratings to eliminate the influence of issue I. According to the previously mentioned analysis result, the rating of a movie with a small distance to movie *β* will have more influence on user *i*. Thus, we introduce a weight for the rating value, based on the distance.

**Fig 9:**
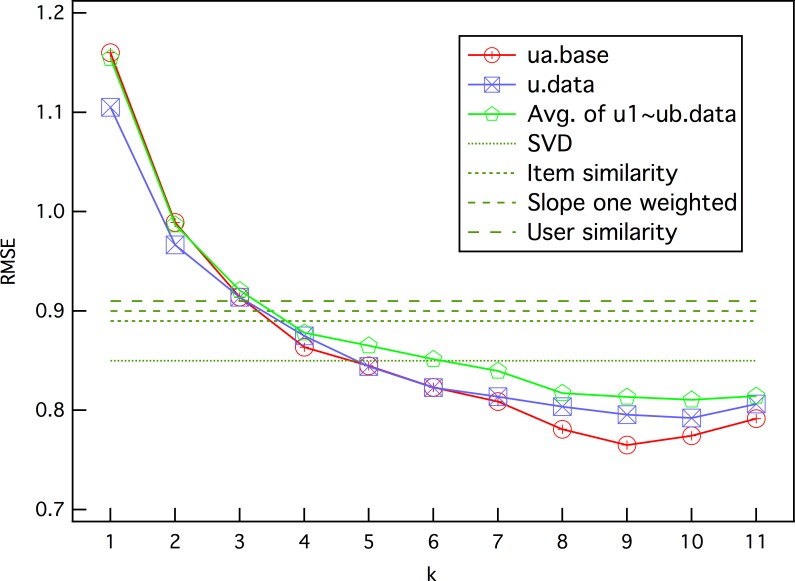
The RMSE of prediction as a function of parameter *k*. *k* is the number of h-neighbors used for prediction.

Fig 9 gives the results of applying the presented user preference model to *MovieLens* data set ua, which has a test data set ua.test with exactly 10 ratings per user. The result data are stored in S4 File, S5 File, S6 File, S7 File and S8 File. To compare it with other typical recommendation algorithms, we use the RMSE as the evaluating indicator of the prediction accuracy.

The red curve in Fig 9 shows that the *k* ≤ 2 prediction error is large, which reflects a difference of opinion between users when they face similar movies. After that the ratings of more similar movies are taken into account, the prediction error of the presented model decreases. The green dashed lines in Fig 9 are the best RMSE values of four typical algorithms [49–52] used for the same data set [1]. When *k* ≥ 5, the presented model can obtain a smaller error of *RMSE* ≤ 0.8447.

Increasing *k* means more data are used to predict the user’s opinion. By common sense, this will continuously enhance the prediction accuracy. However, the result in Fig 9 implies that there is a limit. When *k* is small, adding data is helpful for increasing the prediction accuracy. When *k* > 9, for data set ua, the prediction error begins to increase. This agrees with the phenomenon of the saturation of the prediction power mentioned in ref. [14]. We also checked other data sets of *MovieLens*, including u1~u5, which are 80%/20% splits of the u.data into ux.base and ux.test, and all the test data sets were disjointed. The results indicate that there is a limit in each data set with *k* ∼ 6 − 9, as shown in data in S9 File.

To further test the existence of this limit, we used data set u.data to calculate the distance matrix of the movies, which contained all the rating data, including the test data in ua.test. The blue curve in Fig 9 shows the results, which show an interesting phenomenon that more data can decrease the prediction error only when *k* ≤ 3. After that, a prediction based on the complete data set does not exhibit better prediction accuracy, and may even be worse.

One possible reason for the existence of the prediction limit is that more rating values for movies with longer distances are included in the prediction when *k* becomes larger, which brings useful information and more noise at the same time. Thus, when the data noise is large enough, the benefit of more data will never be notable. The results on data set u.data can prove this even more: more data brings a larger prediction error with the same *k* value when *k* ≥ 3.

**Fig 10:**
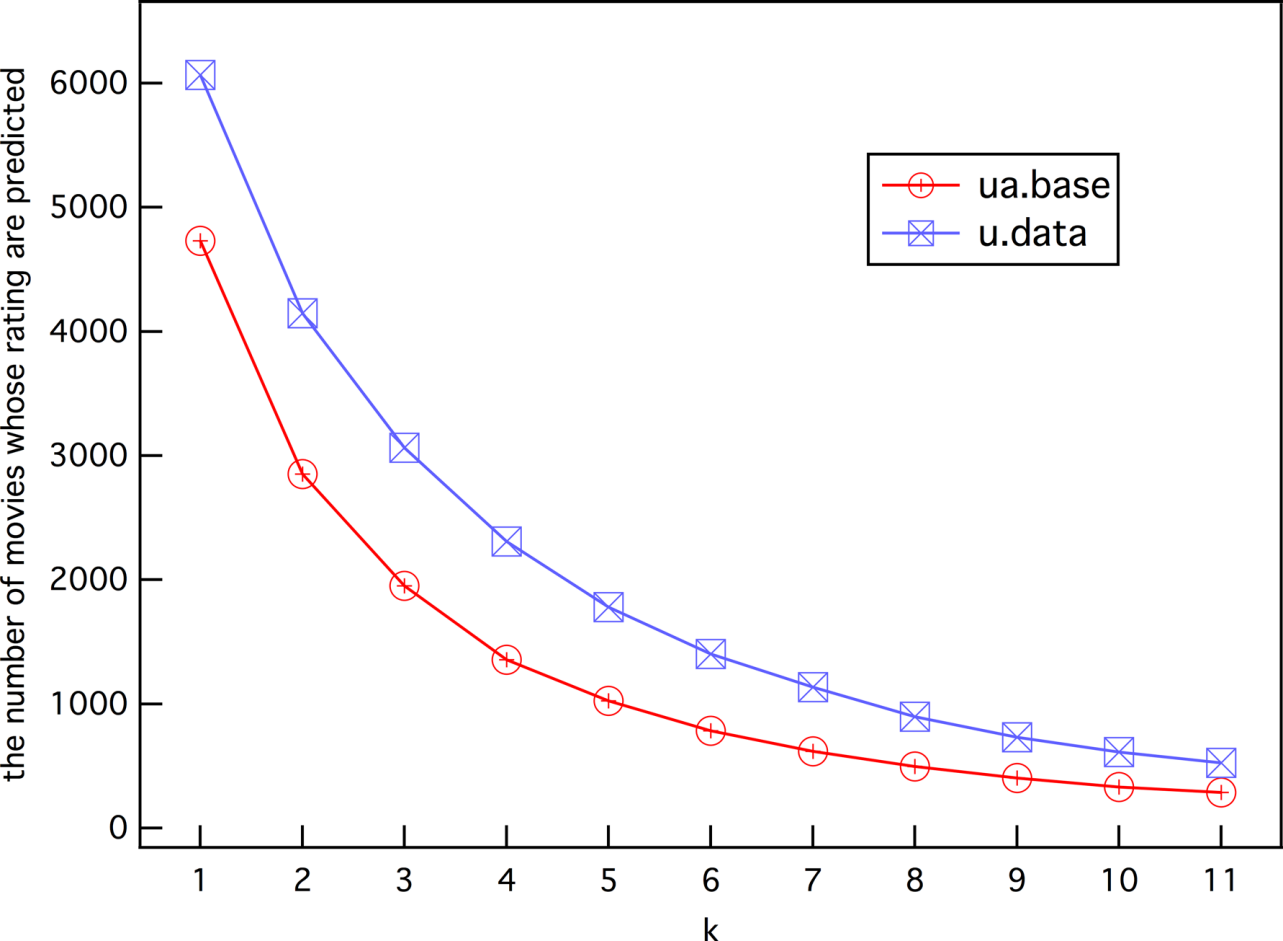
The number of predictable ratings vs. *k* value.

The results in Fig 9 were obtained under the condition of *k* contiguous h-neighbors, which means that the prediction will be discarded once the *x*th (*x* ≤ *k*) h-neighbor has no rating from predicted user *i*. We have investigated in detail how the value of *k* affects the prediction results, as shown in Fig 10. The plots indicate that with increasing *k*, the number of predictable ratings decreases. This illustrates that, for prediction ϒ_*i*_(*β*), an increasing number of movies *m* ∈ *M* have no ratings from user *i* when *k* is larger. The results on the complete data set u.data (blue curve) contain about 1000 more predictable ratings with the same *k*, which further proves the analysis. On the other hand, this result also implies that the prediction accuracy can be improved by using a sufficient amount of useful information–the ratings for the h-neighbors of *β* from user *i*.

Based on the above analysis, we introduce another parameter *η* for controlling the depth of the data used for the prediction. With *η*, the h-neighbors retrieving rule defined in Eq 8 becomes,

Here, *l* = *k*^'^ + *η*^'^ h-neighbors are taken contiguously from *M* until *k*^'^ = *k* or *η*^'^ = *η*, as shown in Eq 9, where *k*^'^ is the number of movies with ratings from user *i*, and *η*^'^ is the number of movies without ratings from user *i*. If the condition *η*^'^ = *η* is satisfied first, the prediction is discarded.

$$x\leftarrow [\overset{k^{'}=k}{\overset{︷}{\overset{↓}{m_{1}},\overset{↓}{m_{2}},\underset{\eta^{'}\leq \eta }{\underset{︸}{\underset{↑}{m_{3}},\overset{↓}{m_{4}},\underset{↑}{m_{5}},\underset{↑}{\cdots_{}}}}\;\overset{↓}{\cdots }}},m_{n}]$$ (9)

After adding *η*, Eq 8 can be expressed in the form

$${ϒ}_{i}(\beta )=\frac{\sum_{x}r_{ix}d_{x\beta }^{-1}}{\sum_{x}d_{x\beta }^{-1}},\;x\;\tilde{\in }\;M=\left\{m|\;\underset{m,\;k,\;\eta }{\text{arg max}}(d_{m\beta })\right\}$$ (10)

**Fig 11:**
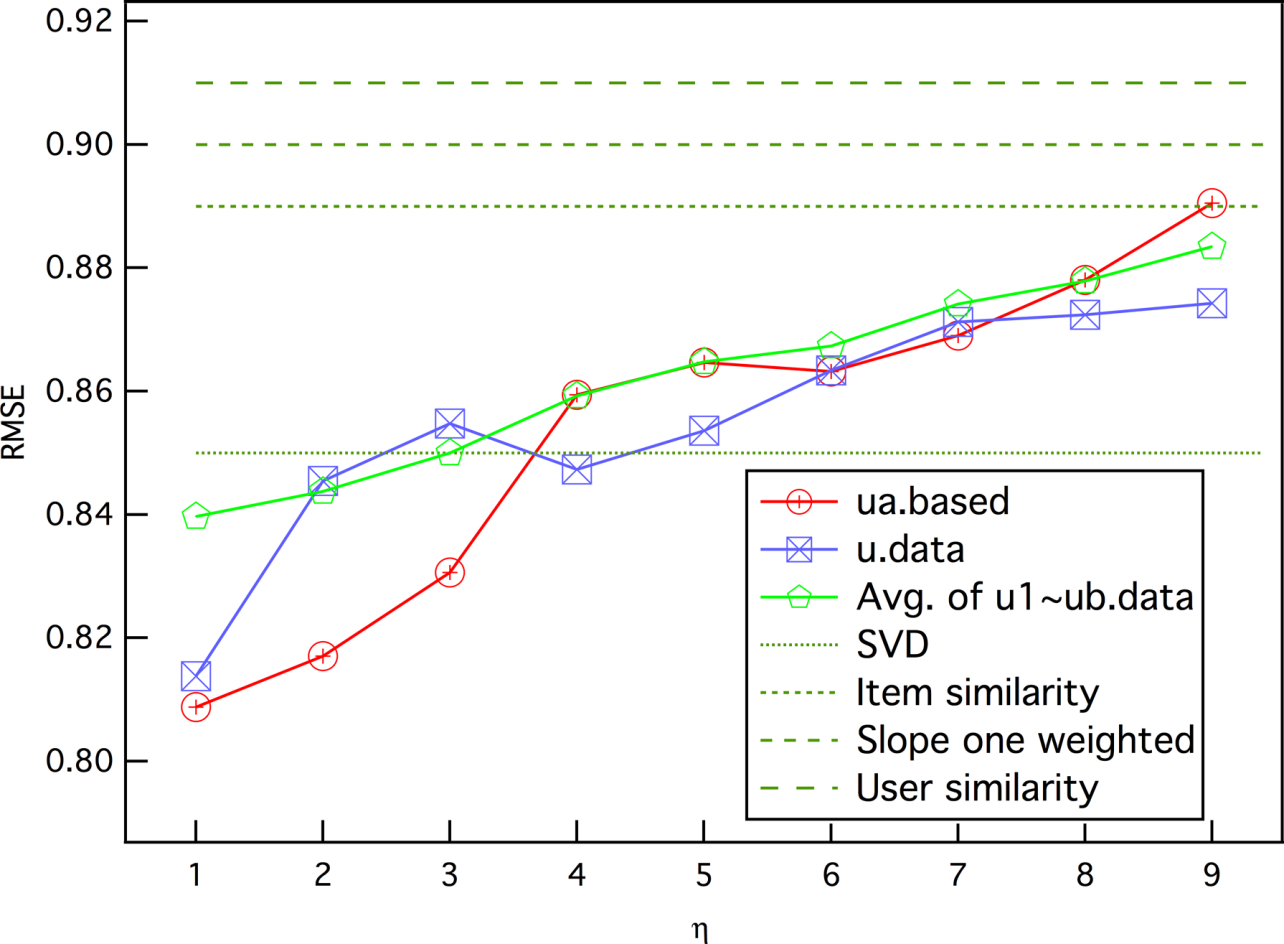
The RMSE of prediction as a function of parameter *η*. *η* is the maximal number of h-neighbors without rating from predicting user *i* before *k* h-neighbors are received. The results are with *k* = 7.

Fig 11 plots the results of the prediction accuracy with parameter *η*. The curves indicate that the RMSE will obviously rise when the ratings of movies with greater distances are considered. The fluctuation illustrates that *η* brings more random factors to the results.

**Fig 12:**
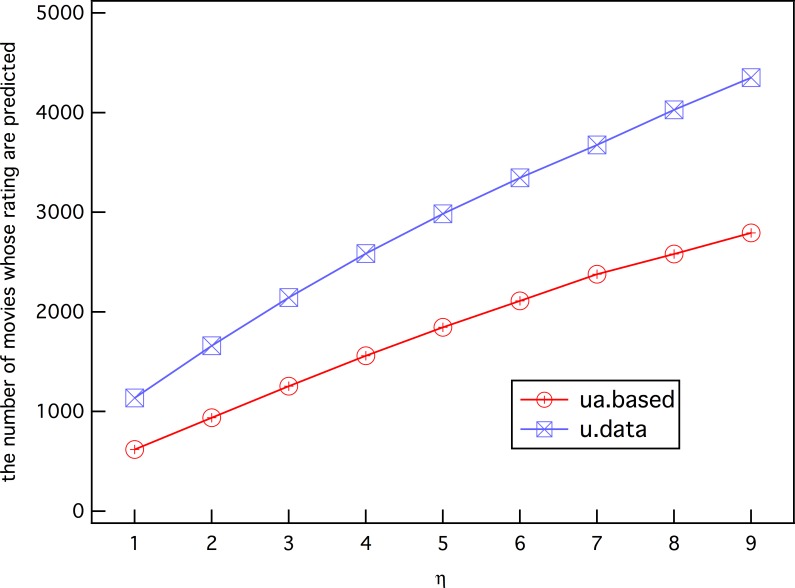
The number of predictable ratings vs. *η* value.

Another result that can be expected is that *η* will reduce the number of discarded predictions, as shown in Fig 12. In other words, *η* has the function of controlling the prediction recall. Clearly, the prediction test on the complete data set u.data has a higher recall than that on ua.base because additional data increase the opportunities for obtaining *k* h-neighbors before *η*^'^ > *η*.

**Fig 13:**
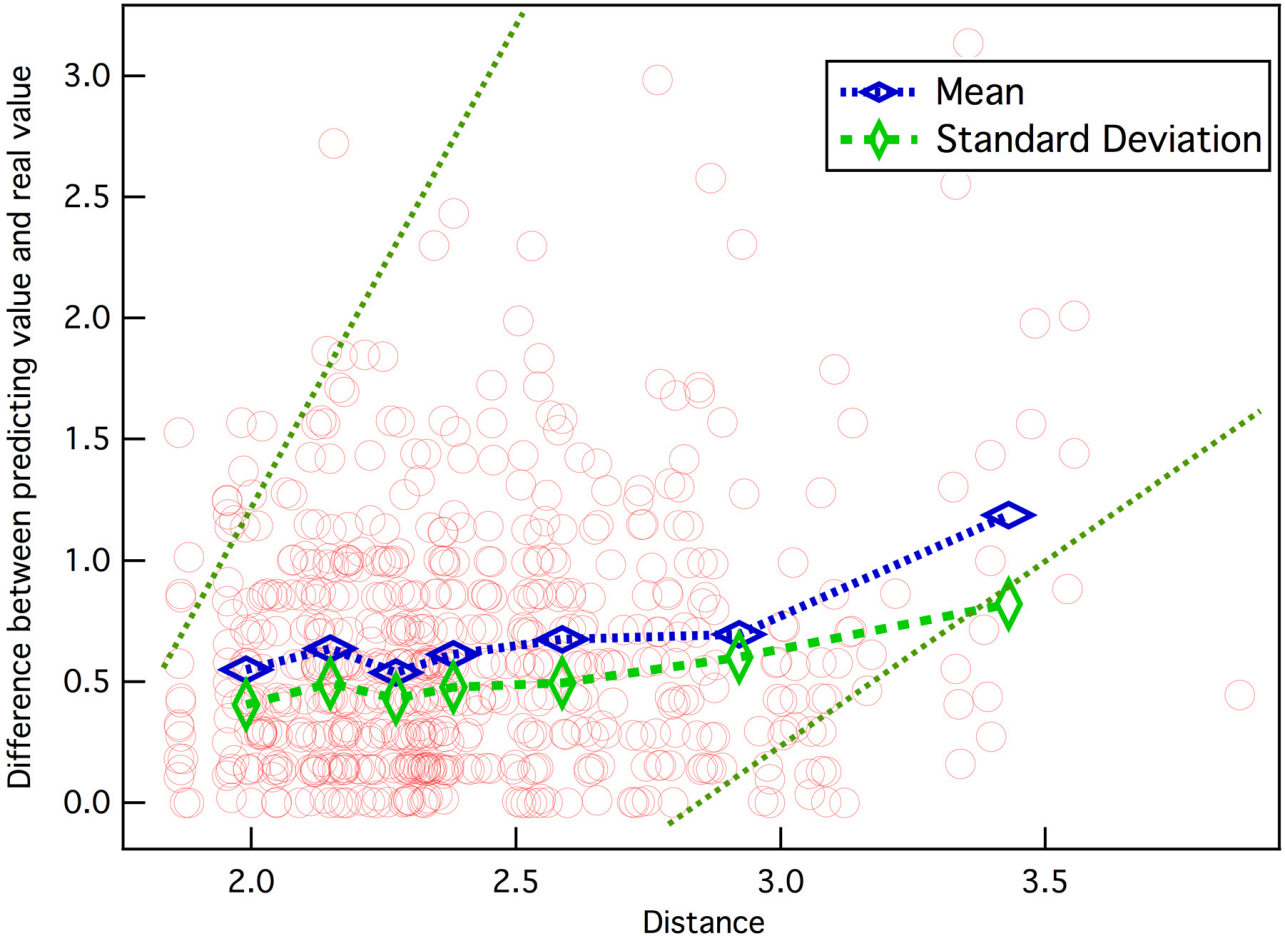
Difference between predicting value and its real value vs. average distance between movies.

We also investigate the direct influence of the neighbor distance on the difference between the predicted value and the real value. Fig 13 shows the results, in which the distance of each point is the mean value of *k* = 7 h-neighbors and *η* = 1. We can note that most points lie in the area with a distance of ~1.8–2.4 and difference of ~0–1.0, and the difference shows the growth trend with increasing distance. The curves of the mean and standard deviation of the difference apparently account for this, and also prove the above analysis. Thus, one can well imagine that, with enough closer movies, the prediction difference could be effectively reduced.

However, in a real system, some users give random ratings at times, and the ratings obtained for movies are always disproportionate. The left parts of the mean curve and standard deviation curve indicate that notable prediction errors still exist even when the average distance between movies is small. As mentioned in [53], the prediction error can never be zero. Thus, it is remarkable that the presented model can eliminate the predictions that are considered to be impracticable. Furthermore, researches about big data also imply that the prediction error would be further reduced by combining the historical data based prediction with other near-real-time data, such as feedback of users [54, 55].

## Conclusions

We investigated one of the famous benchmark data sets–*MovieLens*, using an empirical method. There have been numerous studies on recommendation algorithms. Our purpose was not to construct a new recommendation algorithm, but to attempt to find some potential regularity, give user preference a description, and then discuss what factors affect the prediction results and how to eliminate impracticable predictions.

We first mapped the users and movies into a bipartite hyper-network using the rating data, and then presented a definition of the distance between movies. In this definition, we introduced two factors, the shrinking factor and stretching factor, to overcome the data-scale issues. We studied the bipartite hyper-network and found that movies can form many close cliques when only the first-order h-neighbors are considered, which shows that users have explicit preferences. We also found that the size of these cliques closely follows a power law, which implies that the numbers of ratings received for movies are inhomogeneous.

We statistically analyzed the rating distribution of movies that form two-member cliques, and found that most users actually have similar opinions on such movies. We further investigated the distribution of the distances between many two-movie pairs in the data set. We found that the distance data could be used to describe user preferences and predict their ratings.

Then, based on these analysis results, we introduced a user preference model with two tunable parameters. Test results indicated that the presented model could reflect a user’s preference and obtain prediction results with remarkable accuracy under the condition of compromising on recall. This also implied that the presented model has the ability to determine whether a prediction is impracticable.

Further data analysis illustrated that the distance between movies is crucial to a user’s opinion prediction. It contains information about the user’s preferences. However, random factors in the data make prediction error inevitable. Thus, it becomes very meaningful to distinguish which predictions can be made more accurate.

In this paper, we have only reported a few statistical characteristics of a limited data set, and introduced some preliminary methods. In the future, we hope to analyze more data to examine the universality of our findings and try to find more regularity in user preferences.

## Supporting Information

S1 File User distance matrix and rating matrix of data set u. (ZIP)

S2 File Nearest k-1 neighbors and adjacency matrix of data set u. (ZIP)

S3 File Nearest k-2 neighbors and adjacency matrix of data set u. (ZIP)

S4 File Rating distribution of item and user in data set u. (ZIP)

S5 File Common rating numbers and item similarity of data set u. (ZIP)

S6 File User distance matrix and rating matrix of data set u1. (ZIP)

S7 File Rating distribution of item and user in data set u1. (ZIP)

S8 File Common rating numbers and item similarity of data set u1. (ZIP)

S9 File RMSE and their average value with different k value. (ZIP)
