# Correction: Empirical Study of User Preferences Based on Rating Data of Movies

**DOI:** 10.1371/journal.pone.0152350

**Published:** 2016-03-21

**Authors:** YingSi Zhao, Bo Shen

There are errors in the Funding section. The correct funding information is as follows: This work was supported by the National Natural Science Foundation of China under Grant 61172072, 61271308 (URL: http://www.nsfc.gov.cn/), the Foundation for the Talents under Grant B15RC00020 (URL: http://kxjsc.bjtu.edu.cn/kyxm/index.htm) and Young Social Science Talent Project of Beijing Federation of Social Science Circles under Grant B15M00070 (URL: http://www.bjskl.gov.cn/).
